# Signatures of selection with cultural interference

**DOI:** 10.1073/pnas.2322885121

**Published:** 2024-11-18

**Authors:** Laurel Fogarty, Sarah P. Otto

**Affiliations:** ^a^Department of Human Behavior, Ecology and Culture, Max Planck Institute for Evolutionary Anthropology, 04103 Leipzig, Germany; ^b^Biodiversity Centre and Department of Zoology, University of British Columbia, Vancouver, BC V6T 1Z4, Canada

**Keywords:** gene–culture coevolution, hitchhiking, selective interference

## Abstract

Human evolution is intricately linked with culture, which permeates almost all facets of human life from health and reproduction, to the environments in which we live. Nevertheless, our understanding of the ways in which stably transmitted, evolutionarily relevant human cultural traits might interact with the human genome is incomplete, and methods to detect such interactions are limited. Here, we describe some rules of cultural transmission which could pertain to both humans and cultural nonhuman animals that could lead to the formation and maintenance of stable associations between cultural and genetic traits. Next, we show that, in the presence of such associations, a process analogous to genetic hitchhiking is possible in gene–culture systems. These could leave signatures in the human genome similar to, and perhaps indistinguishable from, those left by selection on genetic traits. Finally, we model selective interference between cultural and genetic traits. We show that selective interference between a cultural trait under selection and a genetic trait under selection can reduce the efficacy of natural selection in the human genome, both in terms of the probability of fixation of beneficial alleles and the dynamics of selective sweeps. We then show that the efficiency of selection at genetic loci can, however, be increased in the presence of strong cultural transmission biases. This implies that the signatures of gene–culture interactions in genetic data may be complex and wide-ranging in gene–culture coevolutionary systems.

## Significance

Human evolution reflects the strong interplay between genes and culture. Here, we explore how cultural processes can alter the signatures of selection left in genomes. We also show how cultural traits can influence the efficiency of selection at genetic loci. These models demonstrate the impact that culture has on genetic evolution, affecting the interpretation of genomic data.

Tests for recent or ongoing natural selection in modern humans have revealed that much recent human evolution is linked to, or the direct result of, human cultural practices such as small-scale horticulture (1), larger-scale agriculture (2, 3), or medical advances (4). Although the cultural underpinnings of many such examples are considered hallmarks of human evolution and carefully described, they are often treated as peculiar aspects of the human environment. However, human culture is best described, not as a part of the human environment, but as a system of inheritance in its own right—a system in which nongenetically encoded information can be passed between and within generations, profoundly affecting human behavior, built environments, and technological capacities (5–7). The hominid archaeological record suggests that the stable transmission and accumulation of cultural information have been features of the human lineage for over 2 million years (8). Because of the pervasive and powerful effects of culture in every known human population, over both ecological and evolutionary timescales, human evolution can only be understood as an interaction between two deeply intertwined inheritance systems: genetic and cultural.

Many frequently cited examples of gene–culture coevolution involve gene–culture fitness epistasis (hereafter, gc-epistasis). Here, the fitness effect of an allele at a genetic locus is altered by the presence of a vertically or obliquely transmitted cultural practice (see section entitled *Cultural Transmission*). Examples of this type of interaction include the coevolution of dairy farming and genes for adult lactose absorption (see e.g., refs. 9–11) and the coevolution of slash-and-burn horticultural practices and the hemoglobin HbS gene (see e.g., ref. 1). In both of these examples, the spread of new modes of production changed the balance of selection pressures at particular loci in the human genome leading to changes in gene frequency.

Fitness gc-epistasis can come about in a number of ways. In the example of the HbS gene, new horticultural methods altered the fitness impacts of different hemoglobin alleles. Hemoglobin is an important oxygen transportation molecule. Alterations in one of its constituent proteins (the *β* protein), caused by mutations in the *β*-globin gene, affect the shape and functioning of red blood cells. Individuals who are homozygous for the *A* allele, and with no other mutations, produce fully functional hemoglobin molecules and red blood cells. Individuals who carry two mutant *S* alleles at the *β*-globin locus frequently produce sickle-shaped red blood cells and are susceptible to anemia. Heterozygous individuals carrying *S* and *A* have red blood cells that are more susceptible to sickling than homozygous *AA* individuals but suffer less from anemia than *SS* individuals. Simultaneously, heterozygotes also benefit from some resistance to malaria. For ancestral populations of humans, this may have been of little consequence (1, 3). However, with the innovation and spread of slash-and-burn horticulture, changes to vegetation density and soil properties created standing water, increasing contact between malaria-carrying mosquitoes and human populations. The malarial resistance afforded to heterozygous *AS* individuals then led to an increase in their fitness and, as a consequence, an increase in frequency of the *S* allele. This example of gene–culture coevolution, and other examples where changes in the cultural environment alter selection on a gene, show relatively straightforward signatures in the human genome—namely the rapid change in frequency of culturally associated genetic alleles, leading to partial or complete selective sweeps.

Alongside these gc-epistatic effects on fitness and selection, the work of Cavalli-Sforza and Feldman (5, 12, 13) suggests that the coevolution of cultural and genetic traits might lead to a number of more complex effects beyond those mentioned above. In particular, (13) showed that under vertical transmission and even under certain forms of oblique cultural transmission (for example involving homophily) a kind of “gene–culture disequilibrium” can arise. Here, a genetic allele can become disproportionately associated with a particular cultural trait. In some cases, the link between a gene and a cultural trait can be maintained mechanistically by the rules of cultural transmission (see below). This constitutes a type of “gene–culture pseudolinkage (gc-pseudolinkage)” and raises several interesting possibilities. First, genetic changes at neutral loci may occur by hitchhiking with selectively favored cultural traits, as occurs between linked genetic loci (14). In particular, genetic variation might be lost, on average, at neutral loci that are gc-pseudolinked with cultural traits under selection. Indeed, early modeling by Feldman and Cavalli-Sforza (12) showed that purely phenotypic selection on a cultural trait can cause genetic changes whenever genes influence the details of cultural transmission. Here, we expand upon this deterministic work and investigate hitchhiking of neutral genetic diversity on a cultural selective sweep and the signatures such a process might produce in the genome.

Second, gc-pseudolinkage can lead to “selective interference,” as occurs with linkage among genetic loci, where the effectiveness of natural selection at one locus is reduced by selection at other loci [also known as the Hill–Robertson effect, (15–17)]. This occurs because chance associations that inhibit selection (e.g., mixtures of favored and disfavored alleles on a chromosome) persist for longer and hinder the spread of beneficial alleles and the purging of deleterious alleles. Selective interference has been shown to reduce the fixation probability of beneficial mutations even when there are no fitness interactions between loci (15, 18). Here, we explore the possibility that the efficacy of selection in the genome may be weakened by interference from selection on culturally transmitted phenotypes, leaving a legacy of milder signatures of selection in the human genome.

We begin by describing some rules of cultural inheritance that could lead to the formation and maintenance of stable associations between cultural and genetic traits, even without gc-epistasis. As long as these associations persist, selective interference between a cultural trait under selection and a genetic trait under selection reduces the efficacy of natural selection in the human genome, both in terms of the probability of fixation for beneficial alleles and the dynamics of selective sweeps. We go on to show that the efficiency of selection at genetic loci can, however, be increased under certain circumstances in the presence of gc-epistasis. This implies that the signatures of such processes may be complex and wide-ranging in a gene–culture coevolutionary system.

The uniquely sophisticated cultural abilities of humans suggest that gene–culture interactions may be common. The human genome may be replete with signatures of interactions between the cultural and genetic transmission systems that have not yet been well described or well understood. Here, we begin to address what some of these signatures may look like and suggest avenues for further development.

## Cultural Transmission.

In most cases, genetic information is passed from parents to offspring. Cultural traits, on the other hand, can be passed from one individual to another, and across generations, in a variety of complex ways. For example, culture can spread from parents to offspring through what is known as vertical transmission, from peer to peer through what is known as horizontal transmission or from older unrelated individuals to younger individuals through oblique transmission (6). Many models of cultural evolution formalize these transmission pathways and work to understand the evolutionary consequences of such varied “transmission modes” (e.g., refs. 19 and 20).

Drawing on research from the fields of animal social learning (21, 22) and anthropology (e.g., refs. 7 and 23), the field of cultural evolution is also concerned with the effects of social learning biases. These are generally conceived of as individual-level cognitive biases that influence when and from whom an individual might gain cultural information. Examples of these include frequency-dependent biases like conformity (disproportionately copying the majority) or anticonformity (disproportionately copying the minority), novelty bias (e.g., refs. 7 and 24) or copying based on the personal characteristics of potential role models such as their age, apparent success, or prestige (e.g., ref. 23).

The variety and complexity of ways in which cultural traits can pass from person to person are the subject matter of research in the field of cultural evolution. Only a subset of these processes and cultural traits are relevant to genetic evolution. For example, cultural traits with no direct biological fitness effect that are transmitted at random with respect to genotype are not expected to leave detectable traces within the human genome, at least directly, although they may do so indirectly for example through changes to effective population sizes (see also refs. 25–27). However, cultural traits with direct fitness effects may alter which genes are passed on to the next generation and which are lost. Our goal in this paper is to determine when and how gene–culture coevolution might change the genomic signatures of selection.

## The formation and maintenance of gene–culture pseudolinkage.

When particular cultural trait and gene combinations are preferentially inherited together, gc-pseudolinkage emerges. Clearly, this linkage cannot be physical in the same sense as the linkage of genes via chemical bonds. Rather, transmission patterns can maintain associations between cultural traits and particular genes, as if they were physically linked, influencing the evolutionary dynamics of both.

In a biparental system with a discrete dichotomous cultural trait (such as we consider below), not all forms of vertical cultural transmission (6, 20) can lead to the formation of associations between a cultural trait and a genetic locus. This was demonstrated in the case of two cultural traits by Ihara and Feldman (28) and Denton et al. (29), where linkage disequilibrium between two cultural traits was zero even when cultural transmission was predominantly from parents to offspring. Nevertheless, there are any number of processes that might facilitate the maintenance of culture–culture or gene–culture associations, including cultural homophily, genetic inbreeding, or learning biases. Here, we focus on two such processes, inspired by the work of Feldman and Cavalli-Sforza (13) and Boyd and Richerson (7), forms of vertical transmission that we label “affinity bias” and “cultural trait bias.”

To describe affinity-biased transmission, we follow the logic of refs. 13 and 20 and assume that a cultural innovation is more often passed from a parent to offspring if they are genetically similar at a particular locus (e.g., have the same genotype). Sex-biased transmission, where daughters preferentially learn from mothers and sons from fathers, falls into this category, based on genetic similarity at the sex-determining locus. In addition, genes that influence learning style or disposition may affect the transmission of cultural traits from parents to offspring. Potential examples include the coevolution of cultural traits such as stone tool use, whose ease of inheritance might depend on genetically determined physical traits such as hand physiology (e.g., refs. 30 and 31). In his book “The Island of the Colorblind,” Oliver Sacks (32) recounted another example: Complete colorblindness is unusually frequent in the Pingelap Atolls and is associated with the cultural practice of night-fishing, the achromatopsia allele increases visual acuity under low light conditions and so may influence the inheritance of fishing practices.

For cultural trait–biased transmission, we follow the literature on genetically determined cultural transmission biases (e.g., ref. 7, p.135) and assume that a cultural innovation is copied preferentially by individuals with a certain genotype, regardless of the genotypes of their parents. Here, genes play a role in determining the strength of the bias toward or against certain cultural traits. For example, genetically determined sensory biases may determine whether a cultural trait is inherited (e.g., OR6A2 variants underlie the soapy taste of cilantro (33), which would affect copying of cilantro use). In birds, genetic hormonal differences affecting boldness also influence habitat preferences for a novel urban environment (34, 35), although the extent to which that preference is learned was not studied.

Both of these models of vertical transmission can generate sustained associations between a cultural trait and a genetic locus. As cultural traits evolve, these associations can drive correlated genetic changes, whether or not direct selection acts on those genes. We turn next to describing these associations with a neutral gene, turning in later sections to a genetic locus subject to selection.

## Genetic Hitchhiking with a Cultural Trait

We consider a selectively neutral diallelic genetic locus A with alleles *A* and *a* and a cultural trait, which we label C for which there is an ancestral form (*c*) and an innovation (*C*). Trait *C* is selectively favored over *c*, raising the biological fitness of its carriers by *s*_*C*_.

In what follows, we simplify the model by considering haploid genetics, representing phenogenotypes by the combination of genetic and cultural traits that they express (e.g., Ac for carriers of allele *A* and cultural trait *c*). The haploid case provides an approximation to the diploid case with intermediate dominance (13). Analyses in *Cultural Interference in a Genetic Sweep* are applicable to randomly mating populations (18). The fitness of the four possible phenogenotypes is

[1] $$\begin{array}{cc} W_{\mathrm{AC}} & =1+s_{C}\\ W_{\mathrm{aC}} & =1+s_{C}\\ W_{\mathrm{Ac}} & =1\\ W_{\mathrm{ac}} & =1 \end{array}$$

Instead of tracking the frequency over time of each phenogenotype, the dynamics can be more readily analyzed using the transformed variables introduced by Maynard Smith and Haigh (14). Specifically, we track the following three variables at time *t*; *x*: the overall frequency of the beneficial cultural innovation *C*, *Q*: the frequency of allele *A* among individuals practicing the beneficial cultural innovation *C*, and *R*: the frequency of allele *A* among individuals practicing the ancestral trait *c*. The frequencies of the phenogenotypes at any point in time are given in Table 1.

**Table 1.** The four possible phenogenotypes and their population frequencies

Phenogenotype	Frequency
*AC*	*xQ*
*aC*	x(1−Q)
Ac	(1−x)R
ac	(1−x)(1−R)

Finally, individuals are allowed to mate and produce offspring, assuming random mating and normal rules of genetic inheritance for the autosomal locus *A*. The rules of transmission are described in Table 2 for both forms of vertical transmission described above: Affinity-biased cultural transmission is facilitated among genetically similar individuals and cultural trait–biased cultural transmission is biased by the genotype of the offspring and the cultural traits of the parents.

**Table 2.** Possible matings among parents, the frequencies of these matings, and the frequencies of phenogenotypes among the offspring

		Affinity bias: Offspring				Cultural trait bias: Offspring			
Mating	Mating frequency	AC	aC	Ac	ac	AC	aC	Ac	ac
AC × AC	$x^{2}Q^{2}W_{\mathrm{AC}}^{2}/{\bar{W}}^{2}$	1	0	0	0	1	0	0	0
AC × aC	$2x^{2}Q\left(1-Q\right)W_{\mathrm{AC}}W_{\mathrm{aC}}/{\bar{W}}^{2}$	0.5	0.5	0	0	0.5	0.5	0	0
AC × Ac	$2x\left(1-x\right)QRW_{\mathrm{AC}}W_{\mathrm{Ac}}/{\bar{W}}^{2}$	0.5	0	0.5	0	*γ*_1_	0	1-*γ*_1_	0
AC × ac	$2x\left(1-x\right)Q\left(1-R\right)W_{\mathrm{AC}}W_{\mathrm{ac}}/{\bar{W}}^{2}$	$\frac{1-\beta_{1}}{2}$	$\frac{\beta_{2}}{2}$	$\frac{\beta_{1}}{2}$	$\frac{1-\beta_{2}}{2}$	$\frac{\gamma_{1}}{2}$	$\frac{\gamma_{2}}{2}$	$\frac{1-\gamma_{1}}{2}$	$\frac{1-\gamma_{2}}{2}$
aC × aC	$x^{2}{\left(1-Q\right)}^{2}W_{\mathrm{aC}}^{2}/{\bar{W}}^{2}$	0	1	0	0	0	1	0	0
aC × Ac	$2x\left(1-x\right)\left(1-Q\right)RW_{\mathrm{aC}}W_{\mathrm{Ac}}/{\bar{W}}^{2}$	$\frac{\beta_{1}}{2}$	$\frac{1-\beta_{2}}{2}$	$\frac{1-\beta_{1}}{2}$	$\frac{\beta_{2}}{2}$	$\frac{\gamma_{1}}{2}$	$\frac{\gamma_{2}}{2}$	$\frac{1-\gamma_{1}}{2}$	$\frac{1-\gamma_{2}}{2}$
aC × ac	$2x\left(1-x\right)\left(1-Q\right)\left(1-R\right)W_{\mathrm{aC}}W_{\mathrm{ac}}/{\bar{W}}^{2}$	0	0.5	0	0.5	0	*γ*_2_	0	$1-\gamma_{2}$
Ac × Ac	${\left(1-x\right)}^{2}R^{2}W_{\mathrm{Ac}}^{2}/{\bar{W}}^{2}$	0	0	1	0	0	0	1	0
Ac × ac	$2{\left(1-x\right)}^{2}R\left(1-R\right)W_{\mathrm{Ac}}W_{\mathrm{ac}}/{\bar{W}}^{2}$	0	0	0.5	0.5	0	0	0.5	0.5
ac × ac	${\left(1-x\right)}^{2}{\left(1-R\right)}^{2}W_{\mathrm{ac}}^{2}/{\bar{W}}^{2}$	0	0	0	1	0	0	0	1

Mating frequencies include the phenogenotype fitnesses given by Eq. **1** when the **A** locus is selectively neutral or Eq. **10** when selected. Normalizing by the mean fitness $\bar{W}$ among the haploid phenogenotypes ensures that the mating frequencies sum to one. The columns for affinity bias describe the case where offspring copy the cultural trait carried by the more similar genetic parent with probability $1-\beta_{i}$ and that of the less similar genetic parent with probability *β*_*i*_ (*i* = 1 or *i* = 2 for offspring carrying allele *A* or *a*, respectively). The columns for cultural trait bias show the offspring phenogenotypes where *γ*_1_ is the bias shown by *A* offspring toward ($\gamma_{1}>0.5$) or against ($\gamma_{1}<0.5$) the *C* cultural trait, and *γ*_2_ is the bias shown by *a* individuals toward ($\gamma_{2}>0.5$) or against ($\gamma_{2}<0.5$) the *C* cultural trait.

Take, for illustration, the mating between an AC parent and an ac parent, shown in the fourth row of Table 2. By the rules of inheritance, the frequency of *A* among offspring is 50% in both forms (summing the AC and Ac columns). Next, the offspring must learn a cultural trait, and it does so by first choosing or aligning with a parent from which to learn. In affinity-biased transmission, *A*-bearing offspring choose to learn from their more genetically similar *A*-bearing parent with probability $1-\beta_{1}$ and from their other parent with probability *β*_1_. Similarly, *a*-bearing offspring learn from their more similar *a*-bearing parent with probability $1-\beta_{2}$. Thus, in affinity bias, the quantities *β*_1_ and *β*_2_ play the role of “recombination rates,” which measure the degree to which genotypes and cultural phenotypes are reshuffled each generation. The extent of this mixing is allowed to differ between offspring with *A* and with *a* (*β*_1_ and *β*_2_ may differ). In cultural trait bias, *γ*_1_ and *γ*_2_ determine the extent to which *A*-bearing offspring and *a*-bearing offspring, respectively, prefer to learn *C* from their parents (assuming the parents differ in their cultural traits). The rest of the table can be read similarly.

According to these rules of inheritance, the frequency of allele *A* is the same in the parents and offspring of each family, so genetic transmission is unbiased. The frequency of the cultural trait is impacted, however, by a form of cultural transmission distortion. Specifically, offspring in AC×ac families have an overall probability of adopting the *C* cultural trait of $(1-\beta_{1}+\beta_{2})/2$, while offspring in Ac×aC families adopt *C* with probability $(1+\beta_{1}-\beta_{2})/2$. Thus, when $\beta_{1}\neq \beta_{2}$, cultural transmission distortion occurs, where the strength of this drive depends on these parameter values and the relative frequencies of these two families. In cultural trait bias, drive can potentially occur in four family types, with a frequency of *C* among offspring of *γ*_1_ in AC×Ac families, $(\gamma_{1}+\gamma_{2})/2$ in AC×ac and Ac×aC families, and *γ*_2_ in aC×ac families. Thus, transmission distortion will occur unless $\gamma_{1}=\gamma_{2}=1/2$, but the extent of distortion will only depend on locus **A** if $\gamma_{1}\neq \gamma_{2}$. Unless $\beta_{1}=\beta_{2}$ in affinity bias or $\gamma_{1}=\gamma_{2}$ in cultural trait bias, the genetic locus thus acts analogously to a preference locus in models of sexual selection, in that it alters the transmission success of cultural traits in a manner that depends on the transmission rules (Table 2). As a consequence, alleles at the genetic locus that prefer to inherit the selectively favored cultural trait will tend to hitchhike along with that trait, generating a type of indirect selection.

The types of genetic-cultural transmission explored here (Table 2) differ from purely cultural models with a similar structure (e.g., refs. 28 and 29) in two important respects: First, here one locus (A) is genetic. Second, and most importantly, the transmission of the cultural trait depends in some way on the alleles carried at the genetic locus.

### Dynamics for Affinity Bias.

Using Table 2 and noting that the mean fitness equals $\bar{W}=1+xs_{C}$ we obtain the following equation for the frequency of the AC phenogenotype in the next generation:

[2] $$\begin{array}{cc} \;\text{freq}{\left(AC\right)}^{\prime } & =x^{\prime }Q^{\prime }\\ & =\frac{x\left(1+s_{C}\right)\left(Q\left(1+xs_{C}\right)+\beta_{1}\left(1-x\right)\left(R-Q\right)\right)}{{\bar{W}}^{2}}, \end{array}$$

where primes denote values in the next generation. The frequency of phenogenotype aC in the next generation is:

[3] $$\begin{array}{cc} \;\text{freq}{\left(aC\right)}^{\prime } & =x^{\prime }{\left(1-Q\right)}^{\prime }\\ & =\frac{x\left(1+s_{C}\right)\left(\left(1+xs_{c}\right)\left(1-Q\right)+\beta_{2}\left(1-x\right)\left(Q-R\right)\right)}{{\bar{W}}^{2}}. \end{array}$$

Summing these, we obtain an expression for $x^{\prime }$:

[4] $$\begin{array}{c} x^{\prime }=\frac{x\left(1+s_{C}\right)\left(1+xs_{C}+\left(1-x\right)\left(R-Q\right)\left(\beta_{1}-\beta_{2}\right)\right)}{{\bar{W}}^{2}}. \end{array}$$

Finally, dividing Eq. **2** by Eq. **4**, we obtain the frequency of *A* among individuals practicing the new cultural trait in the next generation:

[5] $$\begin{array}{c} Q^{\prime }=\frac{Q\left(1+xs_{C}\right)+\beta_{1}\left(1-x\right)\left(R-Q\right)}{\left(1+xs_{C}\right)+\left(1-x\right)\left(R-Q\right)\left(\beta_{1}-\beta_{2}\right)} \end{array}$$

The same method can be used to write expressions for $R^{\prime }$, the frequency of allele *A* among individuals practicing the ancestral cultural trait *c* in the next generation, giving a dynamically complete description of this system. Note that the results of ref. 14 are recovered when $\beta_{1}=\beta_{2}$, as the analogy with recombination is perfect when cultural mixing rates are the same among all genetic offspring.

### Dynamics for Cultural Trait Bias.

Repeating the same procedure when the genetic loci determine a preference for which cultural trait offspring copy, we get Eqs. **6**–**9**. $Q^{\prime },R^{\prime }$ can be calculated similarly in this case.

[6] $$\begin{array}{ll} \text{freq}{\left(AC\right)}^{\prime } & =x^{\prime }Q^{\prime }\\ & =\frac{x(1+s_{C})\left(xQ(1+s_{C})+\gamma_{1}(1-x)(Q+R)\right)}{{\bar{W}}^{2}} \end{array}$$

[7] $$\begin{array}{ll} \text{freq}{\left(aC\right)}^{\prime } & =x^{\prime }{\left(1-Q\right)}^{\prime }\\ & =\frac{x(1+s_{C})\left(\left(1+s_{C})x(1-Q)+\gamma_{2}(1-x)(2-R-Q\right)\right)}{{\bar{W}}^{2}} \end{array}$$

[8] $$x^{\prime }=\frac{x(1+s_{C})\left(\left(1+s_{C})x+(1-x)(Q+R)\gamma_{1}+(2-R-Q)\gamma_{2}\right)\right)}{{\bar{W}}^{2}}.$$

[9] $$Q^{\prime }=\frac{xQ(1+s_{C})+\gamma_{1}(1-x)(Q+R)}{\left(1+s_{C})x+(1-x)(\gamma_{1}(Q+R)+\gamma_{2}(2-R-Q)\right)}$$

### Numerical Analysis.

**Fig. 1.**
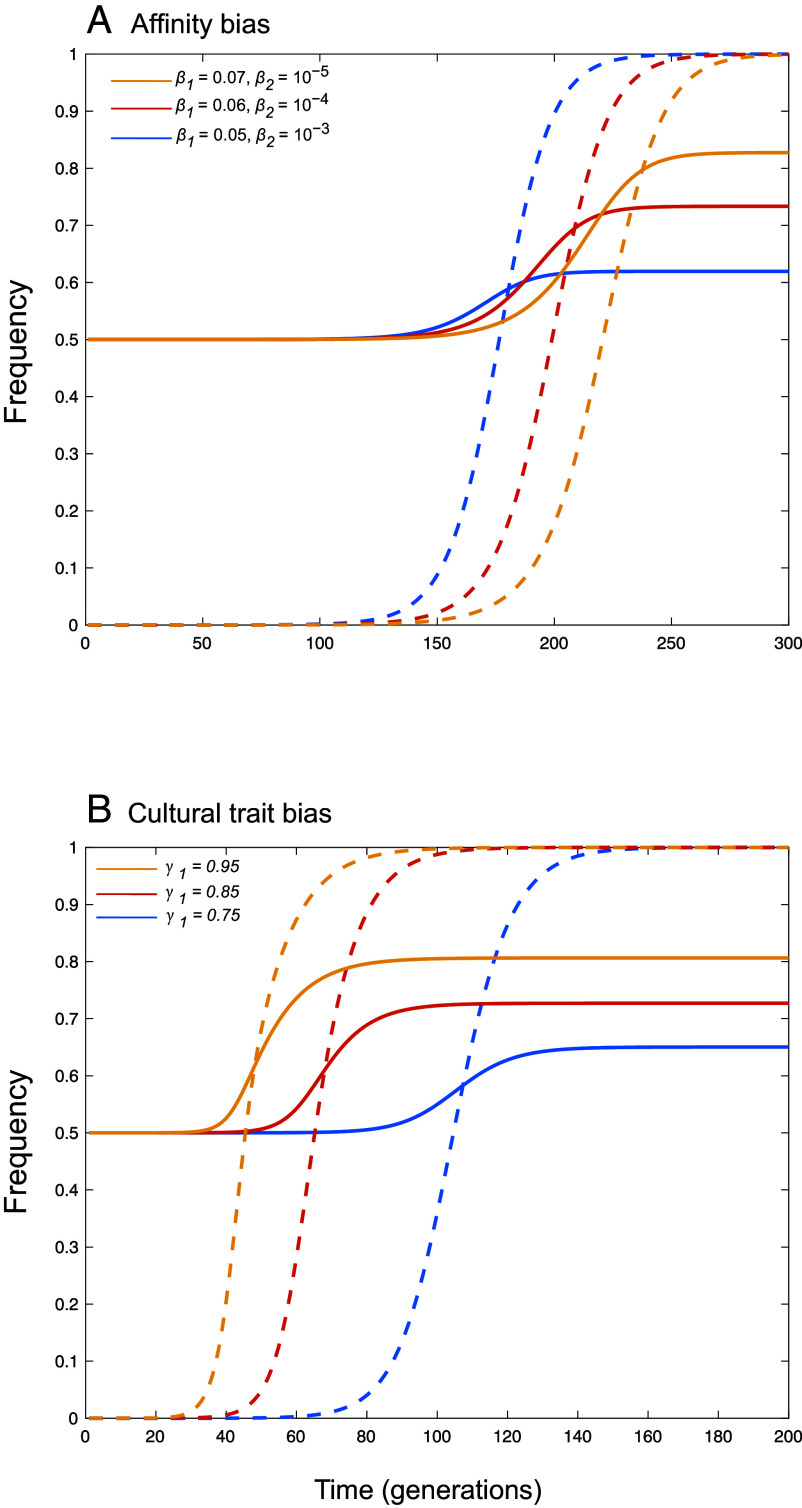
Genetic hitchhiking with cultural traits. A new selectively favored cultural innovation *C* is introduced in a single individual bearing the neutral allele *A*. The rise in frequency of the cultural trait (dashed curves) causes hitchhiking of the neutral allele *A* (solid curves), both in affinity bias (shown in panel *A*) and cultural trait bias (panel *B*) of gene-cultural coevolution considered here. Panel (*A*) shows that hitchhiking is weaker the higher the mixing rates in phenogenotypes, using $\beta_{1}=0.05,\beta_{2}=10^{-3}$ (blue curves), $\beta_{1}=0.06,\beta_{2}=10^{-4}$ (red curves), or $\beta_{1}=0.07,\beta_{2}=10^{-5}$ (yellow curves). Panel (*B*) shows a case where allele *A* tends to copy *C* parents and allele *a* tends to copy *c* parents, using $\gamma_{1}=0.75$ (blue curves), $\gamma_{1}=0.85$ (red curves) or $\gamma_{1}=0.95$ (yellow curves), with $\gamma_{2}=0.5$ in all cases. Other parameters are $N=10^{6}$, $s_{C}=0.1$.

We explore genetic hitchhiking at locus **A**, assuming that the innovation *C* initially arises with allele *A* (without loss of generality, as the allele labels are arbitrary). Simulations show that, for both forms of vertical transmission, hitchhiking occurs, raising the frequency of *A* above its initial value of 0.5 (Fig. 1). For affinity bias, hitchhiking in a gene–culture system is appreciable only when mixing rates are low (particularly *β*_2_), because only then is gc-pseudolinkage tight and gene–culture associations maintained for long periods (Fig. 1*A*). This implies that hitchhiking is possible but there can be little error in identifying and learning from a particular parent. For cultural trait bias, Fig. 1*B* illustrates that hitchhiking can be strong when offspring are biased in the cultural traits that they copy from their parents, depending on the offspring genotype. Here, *a* individuals show no bias ($\gamma_{2}=0.5$) and *A* individuals show a bias toward the beneficial cultural trait.

**Fig. 2.**
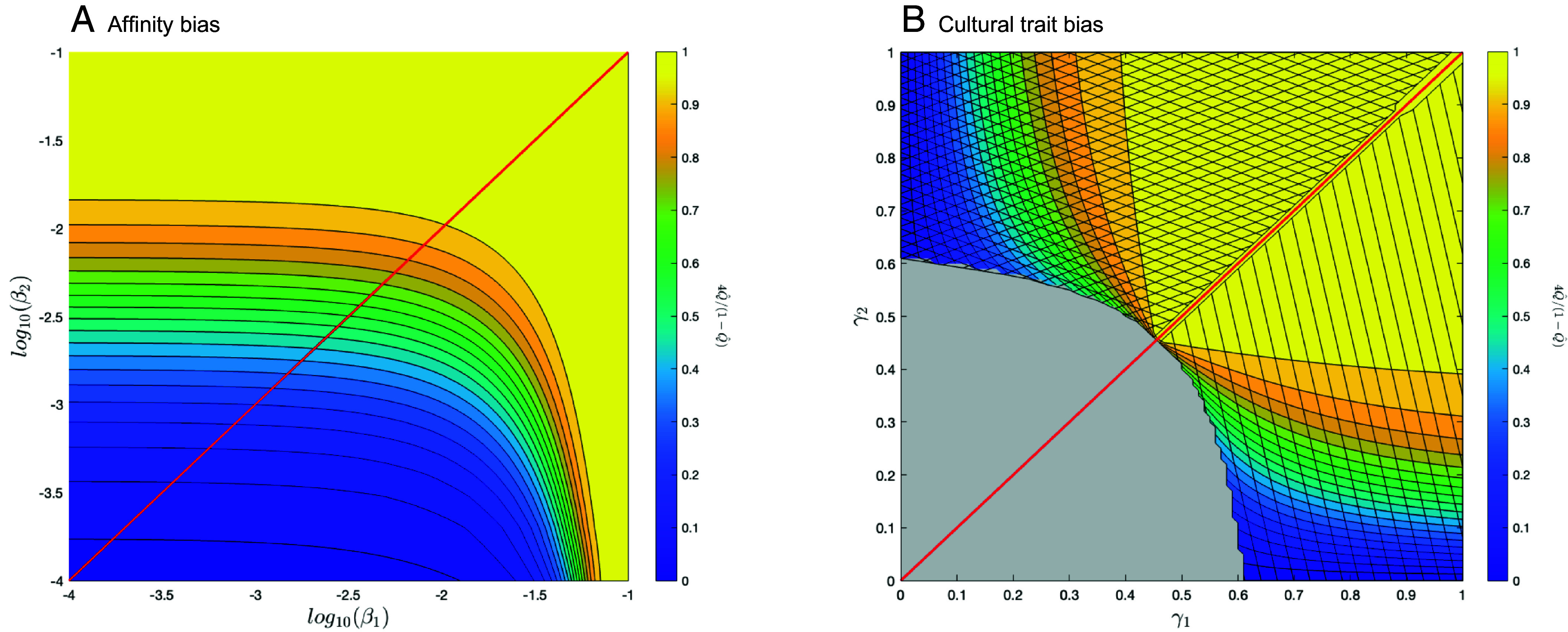
Final relative heterozygosity ($\frac{\hat{Q}\left(1-\hat{Q}\right)}{R_{0}\left(1-R_{0}\right)}=4\hat{Q}\left(1-\hat{Q}\right)$) at a neutral locus **A** after a cultural sweep with affinity-biased (panel *A*) or cultural trait–biased (panel *B*) vertical transmission. The red line in panel (*A*) shows where $\beta_{1}=\beta_{2}$, which is mathematically equivalent to genetic hitchhiking with recombination rate $r=\beta_{i}$ between two genes (14). In panel (*B*), the red line shows where $\gamma_{1}=\gamma_{2}$, the gray area represents parameter range where *C* does not fix. The crosshatched area shows where the final frequency of the *A* allele is less than 0.5, the lined area shows where the final frequency of the A allele is greater than 0.5. Other parameters are $N=10^{6},s_{C}=0.1$.

Fig. 2 shows the final expected heterozygosity (when *C* has either fixed or gone extinct) relative to its initial value, $\hat{H}=\frac{\hat{Q}\left(1-\hat{Q}\right)}{R_{0}\left(1-R_{0}\right)}$ (36), obtained by numerically iterating the above equations. Here, $\hat{Q}$ is the value of *Q*_*t*_ after the cultural sweep has completed (at *t* = 200), and *R*_0_ is the initial frequency of allele *A* among practitioners of cultural trait *c* ($R_{0}=0.5$ in Fig. 2). The effect of the cultural sweep on expected heterozygosity at a genetic locus, *H*, quantifies the impact of cultural hitchhiking on genetic variation with gc-pseudolinkage. A value of one implies that the heterozygosity at the genetic locus has remained unchanged, while lower values indicate that the cultural sweep has reduced variation at the **A** locus.

Because the innovation *C* initially arises with allele *A*, again, we find that hitchhiking effects on variation at locus **A** are strong only when the mixing rate *β*_2_ for affinity bias is very low (Fig. 2*A*, note the log scale). As described in Table 2, *β*_2_ is critical for creating “recombinant” offspring when *C* first appears with allele *A*, allowing the cultural innovation *C* to move from one genetic background to the other (from carriers of *A* to *a*) in families involving AC×ac matings. While higher values of *β*_1_ help drive the new cultural trait *C* into the population (Fig. 1), this has less impact on the extent of hitchhiking.

The red line in Fig. 2 Panel *A* shows where there is equal mixing of cultural traits among offspring inheriting alleles *A* and *a* ($\beta =\beta_{1}=\beta_{2}$). This case corresponds mathematically to genetic hitchhiking, as examined by Maynard Smith and Haigh (14), with *β* in place of the recombination rate, *r*. Along this red line, the same signature of hitchhiking at a neutral locus would be observed in the presence of selection on a physically linked genetic locus or selection on a pseudolinked cultural trait.

Hitchhiking occurs over a broad parameter range with cultural trait–biased gc-pseudolinkage (Fig. 2*B*). The reduction in genetic variation at locus **A** is particularly strong when *γ*_2_ is low, so that few *a*-bearing offspring copy the new cultural innovation *C* when it first appears, maintaining the initial association between cultural trait *C* and allele *A* for longer. That is, when *γ*_2_ is low, AC×ac matings rarely produce the “recombinant” offspring aC. If, however, *γ*_2_ is high and *γ*_1_ is low, offspring bearing *a* are much more likely to take up the new trait *C* than are offspring bearing *A*, which generates an overabundance of aC individuals, reversing the initial association created when *C* arose with allele *A* (cross-hatched region). Finally, if both *γ*_1_ and *γ*_2_ are so low that few offspring copy *C*, the innovation may be lost even though it is selectively favored (gray region).

These results highlight the complexity of the interaction between genes and cultural traits, some of which leave strong signatures, some leave weak signatures, and some leave no detectable trace at all.

**Fig. 3.**
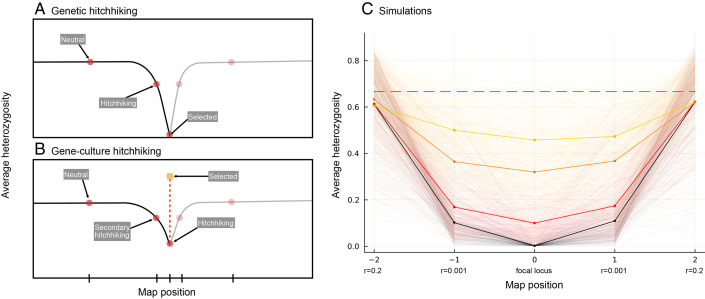
Secondary hitchhiking leads to reduced variation in genomic regions surrounding a gene experiencing gc-pseudolinkage. (*A*) An illustration of genetic hitchhiking. The x-axis represents chromosomal position, the y-axis shows average heterozygosity. Genetic hitchhiking causes a reduction in heterozygosity at neighboring loci. This effect attenuates with genetic distance from the selected locus. (*B*) An analogous illustration of gene–culture hitchhiking. Again, the x-axis represents the chromosome, and the y-axis represents heterozygosity. Here, it is the cultural trait, illustrated by a yellow square, that is under selection. Gc-pseudolinkage maintained by vertical cultural transmission rules (represented by a red dashed line) allows hitchhiking leading to a reduction in diversity at the hitchhiking locus. Genetic linkage then leads to secondary hitchhiking producing a pattern that is qualitatively similar to that in panel (*A*). Panel (*C*) shows simulations of both genetic hitchhiking (black) and gene–culture hitchhiking (colored lines). Five genetic loci are arranged on the x-axis, with one focal locus **A** at map position 0 and four linked loci, two on either side at a recombination distance *r* = 0.001 and two at *r* = 0.2. In all cases, the simulation was run to a steady state for 5*N* generations and then a new beneficial allele was introduced at map position 0 (black curve with $s_{A}=0.1$) or a new favored cultural trait was introduced with gc-pseudolinkage to map position 0 (colored curves with $s_{C}=0.1$, but no selection on locus **A**, $s_{A}=0$), tracking these favored traits until fixation or loss. Stronger hitchhiking is observed with lower rates of genetic mixing (lower *β* values): *β* = 0.001 (red), *β* = 0.005 (orange), *β* = 0.01 (yellow), with $\beta_{1}=\beta_{2}$. Other parameters are N=10,000, mutation rate *μ* = 0.0001 (no mutation at locus **A**). Only cases where the favored trait successfully swept to fixation are shown, with at least 200 independent sweeps shown per condition. The red dashed line shows the expected neutral heterozygosity at freely recombining loci.

While we have only considered one genetic locus above, changes caused by hitchhiking at locus **A** can, in turn, cause nearby genetic loci to change in frequency, mimicking signatures of selective sweeps (37) (Fig. 3 *A* and *B*). The “secondary hitchhiking” caused by a cultural sweep can leave a broad genomic signature of selection, as illustrated in Fig. 3*C* using a simple agent-based simulation of five genetic loci and one cultural trait (details in *SI Appendix*, section S3.1). Even though there is gc-pseudolinkage only between the cultural trait and a single locus (at the central position 0), the average heterozygosity is reduced along the entire stretch of DNA simulated. The purely genetic case, where the genetic locus at 0 is under selection is included for comparison (shown in black). Stronger pseudolinkage between the cultural trait and the genetic locus leaves stronger signatures of selection, both on the gc-pseudolinked locus and surrounding loci (decreasing *β* values of 0.01 in yellow, 0.005 in orange, and 0.001 in red, with $\beta_{1}=\beta_{2}$.) Thus, patterns of low diversity that might typically be interpreted as signifying a genetic selective sweep could, instead, be caused by gene–culture interactions.

**Fig. 4.**
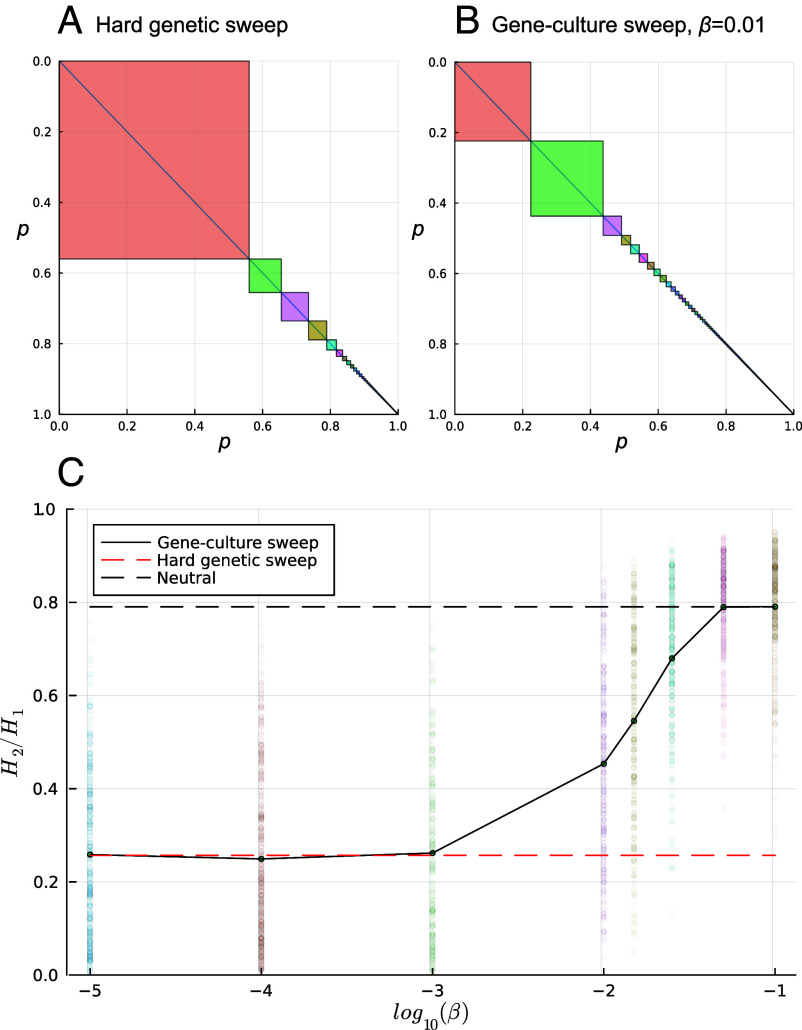
Signatures of hard versus soft selection following gene–culture hitchhiking. (*A* and *B*) Squares show haplotype frequencies following the selective sweep of a favorable cultural trait at five loci, two at recombination distance *r* = 0.01 and two at *r* = 0.001 with respect to the central focal locus causing affinity-biased transmission (see simulation details in *SI Appendix*, section S3.1). (*A*) With perfect copying (*β* = 0), a hard genetic sweep causes one haplotype (*H*_1_) to be much more frequent than all others. (*B*) With imperfect copying (*β* = 0.01), mixing causes more than one haplotype to be at high frequency (here, the most frequent haplotype in red and the second-most frequent haplotype in green). These panels illustrate the result from a single simulation. Panel (*C*) shows the ratio $H_{2}/H_{1}$ for hard selective sweeps (red dashed line), neutral simulations (no selective sweep, black dashed line), and cultural sweeps (black solid curve) for different values of $\beta_{1}=\beta_{2}=\beta$. Dark circles are means of at least 200 sweeps, translucent circles are the results of individual simulation runs.

Unless copying of the more similar genetic parent is nearly perfect, we expect secondary hitchhiking to cause a signature of selection that is more akin to a soft sweep than a hard sweep (37–39). This is because mixing between gene **A** and cultural trait **C** preserves genetic variation at the pseudolinked gene (Fig. 2), allowing more than one haplotype to persist even at the center of the sweep region (Fig. 3 *B* and *C*). Thus, tests of selection might suggest that soft sweeps have occurred when the observed patterns are actually driven by secondary hitchhiking with a cultural trait. To demonstrate this, we apply the H2/H1 test of ref. 40 to simulated data with affinity-biased vertical transmission, assuming that the beneficial cultural trait arose once (*SI Appendix*, section S3.1). This test compares the homozygosity excluding the frequency of the most frequent haplotype ($H_{2}=\sum_{i=2}^{n}p_{i}^{2}$) to the homozygosity including the most frequent ($H_{1}=\sum_{i=1}^{n}p_{i}^{2}$), where $p_{1}...n$ is an ordered list of haplotype frequencies such that *p*_*i*_ is the frequency of the *i*th most frequent haplotype. The ratio $H_{2}/H_{1}$ is expected to be lower under hard than soft sweeps (Fig. 4). Fig. 4*C* shows that when copying is nearly perfect ($\beta_{1}=\beta_{2}=\beta$ very low), only one haplotype is likely to persist in the region, and the test infers a hard sweep. When mixing is frequent (*β* very high), $H_{2}/H_{1}$ is indistinguishable from neutral (dashed line). For intermediate rates of copying, however, the signature of selection mimics a soft sweep, despite the beneficial cultural trait arising only once.

## Cultural Interference in a Genetic Sweep

Next, we examine whether selective interference could occur when both the genetic locus and cultural trait are under selection—a cultural equivalent of the Hill–Robertson effect (15). The existence of such effects would have profound implications for our understanding of the ways in which selection, drift, and draft shape the human genome.

We continue to model gene–culture coevolution as above, but now consider that the *A* allele increases fitness by a factor *s*_*A*_, so that the fitnesses of the four possible phenogenotypes are given by:

[10] $$\begin{array}{cc} W_{\mathrm{AC}} & =\left(1+s_{C}\right)\left(1+s_{A}\right)\\ W_{\mathrm{aC}} & =1+s_{C}\\ W_{\mathrm{Ac}} & =1+s_{A}\\ W_{\mathrm{ac}} & =1 \end{array}$$

To explore gene–culture selective interference, we assume that allele *A* appears initially in a single copy in a random individual, where the current frequency of the beneficial cultural innovation, *C*, is given by *x*. We then investigate how the fate and dynamics of the favorable allele *A* are impacted by the cultural trait, even though the cultural trait has no effect on the fitnesses of individuals bearing alleles *A* and *a*.

### Stochastic Simulations.

**Fig. 5.**
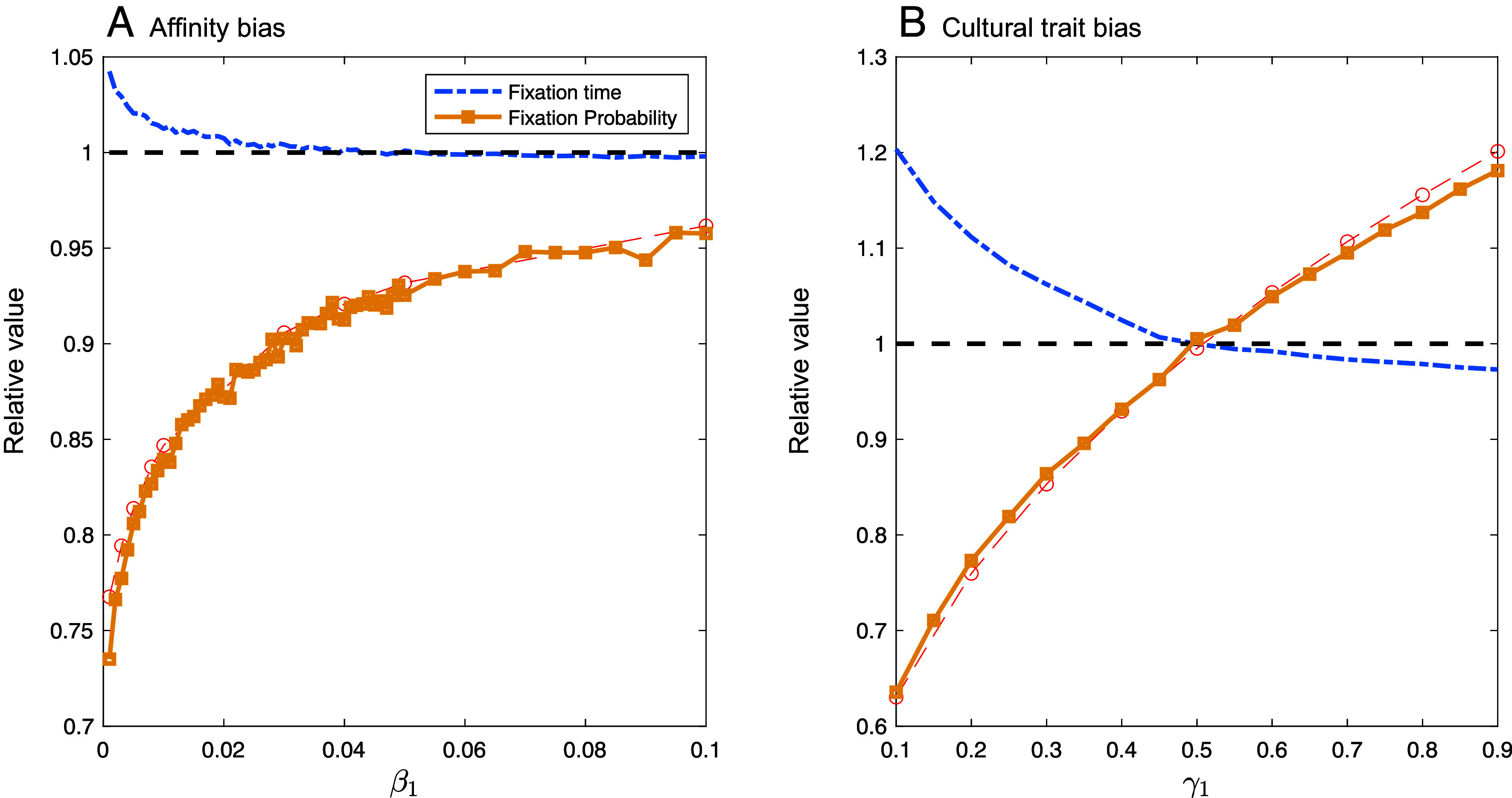
Selective interference caused by a cultural trait transmitted through vertical cultural transmission according to (*A*) affinity bias and (*B*) cultural trait bias. Stochastic simulations of a single locus and a single cultural trait, where the fate of a genetic mutation is tracked after it first appears in a single individual, chosen at random to be practicing the beneficial cultural trait *C* or the ancestral cultural trait *c*. Blue curves show the effect of interference on time to fixation (conditional on fixation). Yellow and red curves show the effect of interference (and associated cultural biases) on the probability of fixation of the *A* allele from simulations and from Eq. **15**, respectively. All quantities are shown relative to their values in the absence of variation in the cultural trait. Further simulation details in *SI Appendix*. Other parameters: $s_{A}=0.1$, $s_{C}=0.1$, $N=10^{3}$, $\gamma_{2}=0.5$, and $\beta_{2}=0.001$.

We conducted stochastic simulations of transmission and selection, as specified in Table 2, within a finite population of size *N*. We explored a range of parameter values, assuming the cultural trait *C* was initially at frequency *x* = 0.5 (see *SI Appendix*, section S3.2 for details). With affinity-biased transmission, Fig. 5*A* shows that selective interference does occur between a cultural trait and a selected locus due to gc-pseudolinkage, causing the beneficial allele *A* to fix less often (orange) and to take longer to fix (blue) than expected in the absence of a cultural trait. These effects become more pronounced when there is less gene-cultural mixing (low *β*_1_ and *β*_2_). Similar results are seen with cultural trait–biased transmission when *A*-bearing offspring are less likely to copy the cultural innovation *C* than *a*-bearing offspring ($\gamma_{1}<\gamma_{2}$; Fig. 5*B*). As discussed in the next section, however, if *A*-bearing offspring are predisposed to inherit the cultural innovation, then cultural selection facilitates rather than interferes with selection at the **A** locus ($\gamma_{1}>\gamma_{2}$; Fig. 5*B*). These results imply that cultural inheritance can change the effectiveness of natural selection on genetic traits and warp the signatures of selection left within the genome, even when gene–culture interactions do not affect fitness.

### Probability of Establishment.

The above simulations demonstrated that interference between selection acting on a genetic locus and that acting on a cultural trait can alter the probability that a new beneficial allele *A* establishes within a population. Here, we build upon the analysis of ref. 18 to derive the probability of fixation of a weakly beneficial genetic mutation (*A*) in the presence of an established, pseudolinked beneficial cultural trait (C:C,c, with frequencies denoted *x* and y=1−x, respectively). Let the fixation probability be *P*_*x*_ when *A* first arises in an individual practicing the cultural trait *C* and *P*_*y*_ when it first arises with *c*. On average, the probability of fixation for a beneficial mutation arising on either background is given by $\bar{P}=xP_{x}+yP_{y}$.

To calculate these probabilities and compare them to the probability in the absence of variation in the cultural trait, we first assume that the mutation *A* arises when the beneficial cultural background has already established and is spreading deterministically. This assumption means that stochastic fluctuations in the cultural trait need not be considered and the frequency of the innovation *C* is approximately:

[11] $$\begin{array}{cc} & x=\frac{1}{1+e^{-s_{C}t}}, \end{array}$$

where *s*_*C*_ is the selective advantage of the cultural trait when allele *a* predominates and time is measured relative to the mid-point of the cultural sweep (*t* = 0 when *x* = 0). For affinity bias, Eq. **11** provides a good approximation to the dynamics. For cultural trait bias, however, biased copying also induces selection on the cultural trait even when allele *a* is nearly fixed. Assuming selection is weak and, thus, that terms $O(s_{C}^{2})$ can be ignored, the overall selection on the cultural trait, accounting for its fitness impact and transmission bias is:

[12] $$\begin{array}{c} {\tilde{s}}_{C}\left(t\right)=s_{C}+2(\gamma_{2}-1/2)(1+s_{C}-2s_{C}x\left(t\right)). \end{array}$$

When $\gamma_{2}=0.5$, there is no transmission distortion among the resident population carrying the *a* allele, and this reduces to ${\tilde{s}}_{C}\left(t\right)=s_{C}$. For illustrative purposes, we set $\gamma_{2}=0.5$ in the following analysis, exploring cases where individuals carrying the new allele *A*, but not the resident allele *a*, are biased in copying cultural traits *C* and *c* from their parents ($\gamma_{1}\neq \gamma_{2}=0.5$).

To find *P*_*x*_ and *P*_*y*_, we use Eqs. **4** and **5** from ref. 18. In the genetic case, these involve the probability that a mutation leaves the background on which it arises, which is given by the rate of recombination multiplied by the probability of the relevant mating. In the case of vertical cultural transmission, the probability that a mutation leaves the *c* background need not be the same as the probability that it leaves the *C* background. Therefore we make a minor adjustment to Eq. **5** of ref. 18 to allow for the possibility that these two rates of ‘jumping’ backgrounds are not the same.

[13] $$\begin{array}{cc} -\frac{\partial P_{x}}{\partial t} & =-r_{1}y\left(P_{x}-P_{y}\right)+\left(s_{A}+s_{C}y\right)P_{x}-\frac{P_{x}^{2}}{2}\\ -\frac{\partial P_{y}}{\partial t} & =-r_{2}x\left(P_{y}-P_{x}\right)+\left(s_{A}-s_{C}x\right)P_{y}-\frac{P_{y}^{2}}{2}, \end{array}$$

where *r*_1_ is the probability that a *A* mutation leaves a *C* background and *r*_2_ is the probability it leaves a *c* background. For affinity-biased transmission, the probability that an *A* mutation leaves a *C* background given the appropriate mating (AC×ac) is the same as the probability that *A* leaves a *c* background among Ac×aC (Table 2); both are given by *β*_1_. Thus for affinity bias $r_{1}=r_{2}=\beta_{1}$. For cultural trait bias, the probability that an *A* mutation leaves a *C* background is $r_{1}=1-\gamma_{1}$ and that it leaves a *c* background is $r_{2}=\gamma_{1}$.

Again following ref. 18, it is possible to describe the mean probability of fixation depending on the cultural background on which the beneficial mutation arose ($xP_{x}+yP_{y}$), and the difference in fixation probability of mutations on the two cultural backgrounds ($P_{x}-P_{y}$). These can be scaled relative to the fixation probability of a beneficial mutation in the absence of hitchhiking, $\;2s_{A}$ (41), to give:

$$\begin{array}{cc} & \Pi =\frac{xP_{x}+yP_{y}}{2s_{A}}\\ & \Delta =\frac{P_{x}-P_{y}}{2s_{A}} \end{array}$$

To solve Eq. **13**, we use the rescaled parameters suggested by Barton (18):

$$\begin{array}{cc} & \rho_{1}=r_{1}/s_{C}\\ & \rho_{2}=r_{2}/s_{C}\\ & \theta =s_{A}/s_{C} \end{array}$$

and we rescale time such that $T=s_{C}t$. Eq. **11** describing the rise in frequency of the cultural innovation *C* then becomes:

$$\begin{array}{cc} & x=\frac{1}{1+e^{-T}}, \end{array}$$

changing over rescaled time according to:

$$\begin{array}{cc} & \frac{\partial x}{\partial T}=\frac{e^{T}}{{\left(1+e^{T}\right)}^{2}}=xy. \end{array}$$

Similarly, the frequency of trait *c* (y=1−x) changes over time according to ∂y/∂T=−∂x/∂T=−xy.

In these rescaled units, we apply the chain rule to obtain ∂Π/∂T:

[14] $$\frac{\partial \text{Π}}{\partial T}=\frac{1}{2s_{A}}\left(\frac{\partial x}{\partial T}P_{x}+\frac{\partial P_{x}}{\partial T}x+\frac{\partial y}{\partial T}P_{y}+\frac{\partial P_{y}}{\partial T}y\right)$$

[15] $$\frac{\partial \text{Π}}{\partial T}=xy(\rho_{1}-\rho_{2})\text{Δ}-\theta \text{Π}(1-\text{Π})+\theta xy{\text{Δ}}^{2}$$

and $\frac{\partial \Delta }{\partial T}$:

[16] $$\frac{\partial \text{Δ}}{\partial T}=\frac{1}{2s_{A}}\left(\frac{\partial P_{x}}{\partial T}-\frac{\partial P_{y}}{\partial T}\right)$$

[17] $$\frac{\partial \text{Δ}}{\partial T}=\text{Δ}\left(\left(\rho_{1}y+\rho_{2}x)+(2\text{Π}-1)\theta +(x-y)(1-\theta \text{Δ}\right)\right)-\text{Π}.$$

**Fig. 6.**
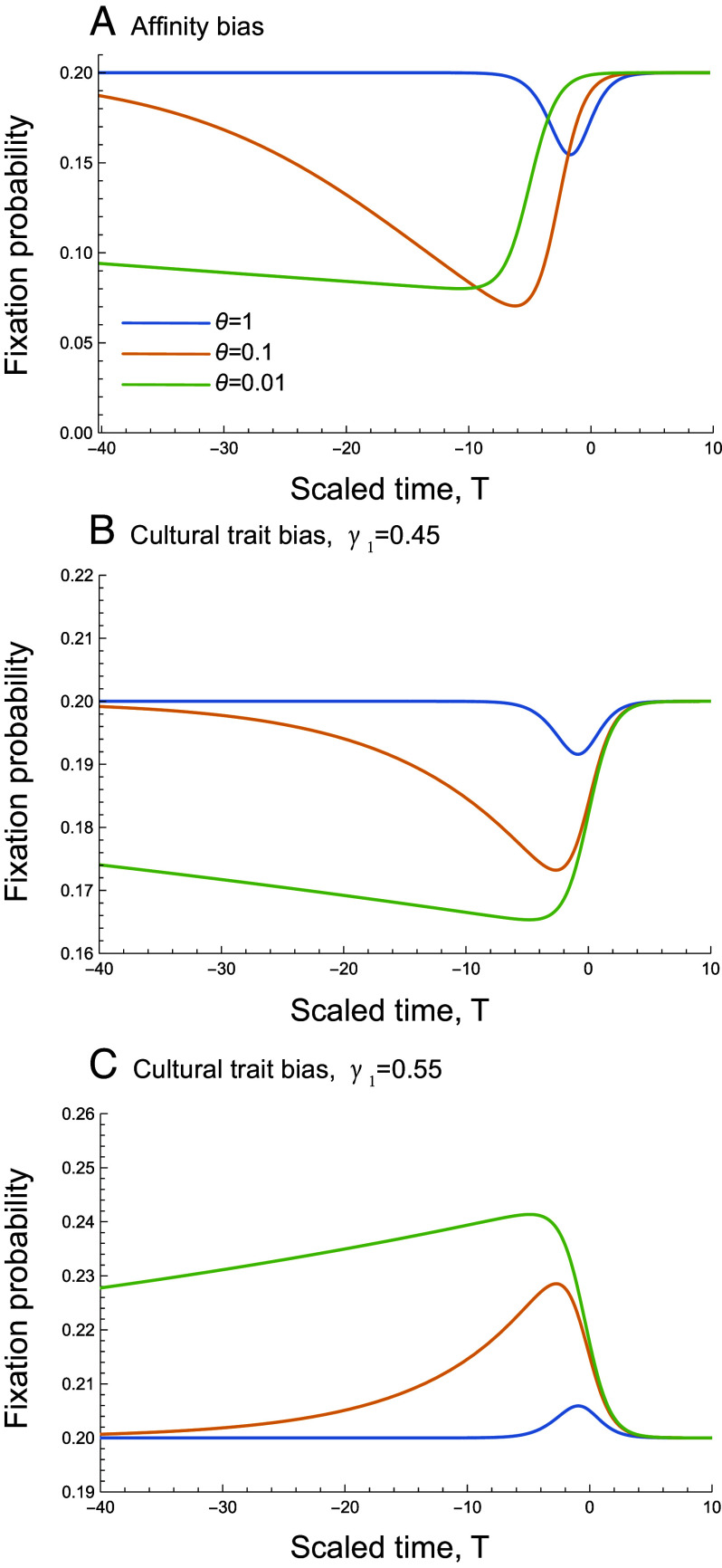
The fixation probability of a beneficial new mutation as a function of the time at which the mutation occurred, scaled by the selective benefit of the cultural background ($T=ts_{C}$) for (*A*) affinity-biased vertical transmission and (*B*) cultural trait–biased vertical transmission with bias against *C* and (*C*) cultural trait–biased vertical transmission with a bias toward *C*. Colored lines show results for different values of $\theta =s_{A}/s_{C}$: blue: *θ* = 1, orange: *θ* = 0.1, green: *θ* = 0.01. Other parameter values are $\beta_{1}=0.01$ (the fixation probability is unaffected by *β*_2_), $s_{C}=0.1$, $\gamma_{1}=0.45$ in panel (*B*) and $\gamma_{1}=0.55$ in panel (*C*), both with $\gamma_{2}=1/2$.

These equations can be solved numerically (see *Mathematica* notebook) to obtain the fixation probability of allele *A* with selective interference from the cultural trait. For affinity-biased transmission, the fixation probability of a beneficial mutant allele with cultural interference is identical to the purely genetic case, with *β*_1_ playing the role of recombination rate *r* (Fig. 6*A*). Only *β*_1_ and not *β*_2_ influences the fixation probability of allele *A* because the establishment of this allele when rare only depends on how *A*-bearing offspring copy the cultural trait practiced by their genetically similar parent. Thus, the approximate analytical solution obtained by Barton (18) for the net effect of the cultural sweep on the fixation probability at locus **A**, considering all possible timings of the cultural sweep, applies equally well to gene–culture selective interference (see his Eqs. **8** and **9**).

For cultural trait bias, the strength and even direction of interference between a cultural trait and gene **A** depends on the biases that occur when copying the parental cultural trait (Fig. 6 *B* and *C*).When *A*-bearing offspring tend to copy the ancestral cultural trait *c*, the fixation probability of *A* declines ($\gamma_{1}<0.5$, panel *B*). When *A*-bearing offspring prefer to copy the innovation *C*, however, allele *A* is more likely to fix, because offspring carrying the selectively favored allele are also more likely to practice the selectively favored cultural trait. In either case, indirect selection acts on locus **A** because copying tends to associate the new *A* allele with whichever cultural trait that *A*-bearing offspring prefer to copy. Thus, phenotypic copying perturbs the dynamics of the selectively favored allele *A*, increasing its rate of spread when copying the selectively favored cultural trait *C* ($\gamma_{1}>0.5$) and decreasing its rate of spread when copying *c* through hitchhiking. While affinity-biased inheritance always generates selective interference that reduces the fixation probability of a beneficial allele *A*, cultural trait bias can lead to an increase or decrease in *A*’s fixation probability, depending on the nature and strength of the cultural transmission bias.

## Discussion

In their now-classic 1973 and 1976 papers, Cavalli-Sforza and Feldman examined the effects of natural selection on the spread of a culturally determined phenotype (e.g., a skill), when learning that phenotype depended on an individual’s genotype. They showed that, even though selection did not act directly on the genes involved, selection for the cultural trait could lead to changes in equilibrium gene frequencies under a variety of selection regimes. This demonstrated that selection on cultural traits could have a profound effect on genetic dynamics. In 1984 Feldman and Cavalli-Sforza went on to show that under vertical transmission, gene–culture disequilibrium—statistical linkage between cultural traits and genetic alleles—could form and be maintained, again with consequences for gene frequencies. Here, we build on this work, formulating two models of vertical transmission that cause gene–culture associations, to investigate the genomic signatures left by selection on cultural traits.

Using these models, we showed that natural selection acting on a cultural trait has the potential to leave a detectable signature in the human genome. In a purely genetic system, selection at a locus is expected to create a pattern of low diversity around that locus (14). We showed that a similar process can lead to a pattern of reduced diversity as a result of selection on the cultural trait alone, even when the genetic loci experience no direct selection. Interpreted in isolation, such a signature (e.g., Figs. 3*C* and 4) would look indistinguishable from a sweep at a gene under selection, particularly soft or incomplete sweeps (37).

One of the most pressing issues for any developing theory of gene–culture coevolutionary hitchhiking or interference is the plausibility of the range of parameters over which these effects are likely to be strong. This relies on estimating the relevant cultural transmission parameters such as the strength of cultural selection (42, 43) and the likely biological fitness effect of cultural traits (44). For example in our affinity-biased vertical transmission, where offspring tend to align with and copy their more genetically similar parent, the values of *β*_1_ and *β*_2_ that allow hitchhiking are very low (Fig. 2*A*). That is, the “mixing” of the genes and cultural traits among offspring must be small relative to the strength of selection on the cultural trait for gene-cultural associations to be maintained over the time period that the cultural trait is segregating within the population. This range of values is feasible in a genetic context because rates of recombination can be low at neighboring loci. However, it is unlikely that offspring will faithfully identify and copy genetically similar parents with little error. We thus conclude that, in the absence of copying biases, most cultural traits are not likely to be transmitted from parents to offspring with sufficiently high fidelity to allow appreciable hitchhiking to occur, unless selection acting on that cultural trait is strong.

On the other hand, genes may often bias cultural inheritance toward one type of cultural trait or another (as in cultural trait–biased transmission). When this occurs, the overall strength of selection, including transmission distortion, is altered by the genes carried by an individual (see Eq. **12**). As the cultural trait evolves, so too will the genes that bias offspring toward copying that cultural trait. This indirect selection is driven by the maintenance of genetic associations built by preferences, here by offspring preferring to learn from one parent over the other, depending on the cultural traits of those parents. As a consequence, the hitchhiking that results from genetic biases in cultural transmission occurs over a much broader range of parameters (Fig. 2*B*).

By scanning genomes for regions where selection appears to be less effective than expected, several authors have found evidence that selective interference has had a substantial impact on the evolution of several species (e.g., ref. 45–47). Here, the strongest effects are found in gene-rich, high mutation, low recombination genomic regions. In the gene–culture case, it is perhaps reasonable to expect the strongest effect of selective interference to be found when innovation rates are high and co-occur with forms of vertical cultural transmission where genes influence the cultural preferences of offspring (exhibiting biased copying as in cultural trait–biased transmission).

Further development of a gene–culture coevolutionary theory of hitchhiking and selective interference would require a better understanding of the distribution of fitness effects of cultural innovations, alongside empirical investigations of the strength and nature of gene–culture pseudolinkage. Development of this theory would also make clear the data, or kind of data, necessary to distinguish gene–culture hitchhiking from genetic hitchhiking. Estimating gene–culture associations within families (akin to looking for disequilibrium between genetic loci) can help identify which cultural traits may be important, allowing researchers to focus on the strength of selection and forms of copying bias that maintain these associations. Temporal data provide an especially powerful means of estimating selection that can be applied to cultural traits (e.g., using Eq. **11** to estimate ${\tilde{s}}_{C}$) and could be combined with genomic data to estimate autocorrelated changes in allele frequencies and cultural traits over time, which is a powerful means of detecting hitchhiking in genomic data (48). Given the rapid pace of cultural evolutionary change (49) it is reasonable to suggest that gene–culture coevolutionary phenomena may be easier to detect in longitudinal data, which is increasingly collected and understood in both cultural (e.g., ref. 50) and genetic (e.g., ref. 51) contexts.

In 2010, Prichard et al. (52) argued that the search for positive selection in the human genome should be broadened to include more than signatures of hard sweeps, including forms of selection leaving less obvious signatures such as soft sweeps and polygenic adaptation. Here, we have argued that this broadening would fruitfully include forms of selection that account for the uniquely potent human capacity to survive and adapt through culture.

Even the most impactful and all-encompassing changes in the cultural lives of ancestral humans are often considered to be special cases of environmental change. However, culture is qualitatively different from any other aspect of the human environment. Culture evolves and so forms a stable and important part of a “dual inheritance” system, which can change the course of genetic evolution in theoretically tractable ways. The development of the theory of cultural evolution and gene–culture coevolution that has occurred over the last fifty years now means that the transmission and maintenance of relevant cultural traits can be understood and integrated into a more complete theory of human evolution.

## Supplementary Material

Appendix 01 (PDF)

## Data Availability

Code data have been deposited in Zenodo (https://zenodo.org/records/13644840) (53).
