# Supplementary material for: Signatures of selection with cultural interference
Source: Proc Natl Acad Sci U S A. 2024 Nov 18;121(48):e2322885121. doi: 10.1073/pnas.2322885121 (PMC11621839; doi:10.1073/pnas.2322885121)
Supplement: Supplementary file 1 — Appendix 01 (PDF) [file pnas.2322885121.sapp.pdf]

Signatures of selection with cultural interference: supplementary material

August 15, 2024

S1. Linkage disequilibrium

As shown by Feldman and Cavalli-Sforza [1984], non-zero gene-culture linkage disequilibrium can be generated by certain forms of cultural transmission.

To illustrate this we show a calculation for affinity biased vertical transmission where just the cultural trait is under selection. To calculate the linkage disequilibrium in the next generation, using values from the current generation (LD'), we first need to write the frequencies of the four phenogenotypes in the next generation in full. these are given by:

$$\bar{W}^2 x_1' = f_1(1+S_C)^2 + f_2\frac{(1+S_C)^2}{2} + f_3\frac{1+S_C}{2} + f_4\frac{1+S_C}{2}(1-\beta_1) + f_6\frac{1+S_C}{2}\beta_1 \quad (1)$$

$$\bar{W}^2 x_2' = f_2\frac{(1+S_C)^2}{2} + f_4\beta_2\frac{1+S_C}{2} + f_5(1+S_C)^2 + f_6(1-\beta_2)\frac{1+S_C}{2} + f_7\frac{1+S_C}{2} \quad (2)$$

$$\bar{W}^2 x_3' = f_3\frac{1+S_C}{2} + f_4\frac{1+S_C}{2}\beta_1 + f_6\frac{1+S_C}{2}(1-\beta_1) + f_8 + \frac{f_9}{2} \quad (3)$$

$$\bar{W}^2 x_4' = f_4\frac{1+SC}{2}(1-\beta_2) + f_6\frac{1+SC}{2}\beta_2 + f_7\frac{1+SC}{2} + \frac{f_9}{2} + f_{10} \quad (4)$$

Using these we can now define LD':

$$LD' = x_1'x_4' - x_2'x_3' \quad (5)$$

$$LD' = \frac{1}{\bar{W}^3}(x-1)x(Q-R)(1+S_C)(1+xS_C+\beta_1(R-1+x(Q-R+(Q-1)S_C))-\beta_2(R+x(Q-R+QS_C))) \quad (6)$$

FigureS1.pdf

Figure S1. Plot of linkage disequilibrium displaying the relationship between LD' and R (first panel) and LD' and the frequency of the cultural trait under selection, x (second panel). With $x = 0.4, R = 0.2, S_C = 0.001, \beta_1 = 0.00001, \beta_2 = 0.00005$, $Q = 0.6$ (blue lines), $Q = 0.7$ (orange lines), $Q = 0.8$ (green lines), unless varied on the x-axis.

FigureS2_LD-eps-converted-to.pdf

Figure S2. Linkage disequilibrium during a sweep where (A) a genetic allele is under selection (from Maynard Smith and Haigh [1974]), (B) a vertically transmitted cultural trait is under selection (affinity bias) and (C) a vertically transmitted cultural trait is under selection (cultural trait bias). Parameter values are the same as Figure 1 from the main text. In panel (A) $\beta_1 = 0.07, \beta_2 = 10^{-}5$ (yellow lines), $\beta_1 = 0.06, \beta_2 = 10^{-}4$ (red lines), $\beta_1 = 0.05, \beta_2 = 10^{-}3$ (blue line). For panel (C) $\gamma_1 = 0.75, \gamma_2 = 0.5$ (blue line),$\gamma_1 = 0.85, \gamma_2 = 0.5$ (red line),$\gamma_1 = 0.95, \gamma_2 = 0.5$ (yellow line). Other parameters are $N = 10^6, S_C = 0.1$.

Note that when $Q = R$, $LD' = 0$.

S2. Alternative presentation

There are a number of ways to present recursions that describe the change in frequency of the phenogenotypes from one generation to the next. In the main text we chose to represent these in a way that makes it easy to track the association between A and C and the difference between this and the association between a and C. However, it is also possible to write these changes in the following way.

As in the main text, the fitness of the four possible phenogenotypes are:

$$W_{AC} = 1 + S_C$$

$$W_{aC} = 1 + S_C$$

$$W_{Ac} = 1$$

$$W_{ac} = 1$$

Next, we track the frequency of each phenogenotype (labelled u_i), in the following way:

Phenogenotype	Frequency
AC	u_1
aC	u_2
Ac	u_3
ac	u_4

Table 1: The four possible phenogenotypes and their population frequencies.

Using these to calculate the frequencies of particular matings (or families), we get the following mating table:

Family	Family frequency	Affinity bias: Offspring				Cultural trait bias: Offspring			
		AC	aC	Ac	ac	AC	aC	Ac	ac
AC×AC	u_1^2	1	0	0	0	1	0	0	0
AC×aC	$2u_1u_2$	0.5	0.5	0	0	0.5	0.5	0	0
AC×Ac	$2u_1u_3$	0.5	0	0.5	0	γ_1	0	1-γ_1	0
AC×ac	$2u_1u_4$	$\frac{1-\beta_1}{2}$	$\frac{\beta_2}{2}$	$\frac{\beta_1}{2}$	$\frac{1-\beta_2}{2}$	$\frac{\gamma_1}{2}$	$\frac{\gamma_2}{2}$	$\frac{1-\gamma_1}{2}$	$\frac{1-\gamma_2}{2}$
aC×aC	u_2^2	0	1	0	0	0	1	0	0
aC×Ac	$2u_2u_3$	$\frac{\beta_1}{2}$	$\frac{1-\beta_2}{2}$	$\frac{1-\beta_1}{2}$	$\frac{\beta_2}{2}$	$\frac{\gamma_1}{2}$	$\frac{\gamma_2}{2}$	$\frac{1-\gamma_1}{2}$	$\frac{1-\gamma_2}{2}$
aC×ac	$2u_2u_4$	0	0.5	0	0.5	0	γ_2	0	$1-\gamma_2$
Ac×Ac	u_3^2	0	0	1	0	0	0	1	0
Ac×ac	$2u_3u_4$	0	0	0.5	0.5	0	0	0.5	0.5
ac×ac	u_4^2	0	0	0	1	0	0	0	1

Table 2: Possible families (including the cultural trait possessed by the parents), their frequencies, and the frequencies of the next generation. The columns labelled 'Affinity bias' show the phenogenotypes in offspring in the case where β_1 is a tendency shown by A offspring to copy a different-genome parent, and β_2 is a tendency shown by a individuals to copy a different-genome parent. The columns labelled 'Cultural trait bias' show the same for the case where where γ_1 is the bias shown by A offspring towards ($\gamma_1 > 0.5$) or against ($\gamma_1 < 0.5$) the C cultural trait, and γ_2 is the bias shown by a individuals towards ($\gamma_2 > 0.5$) or against ($\gamma_2 < 0.5$) the C cultural trait.

Using Table 3, it is possible to write down the phenogenotype frequencies in the next generation as a function of the frequencies in this generation and the linkage disequilibrium, D given by $D = u_1u_4 - u_2u_3$. For affinity bias, these are:

$$\bar{W}^2u_1' = u_1^2(1+S_C)^2 + u_1u_2(1+S_C)^2 + u_1u_3(1+S_C) + u_1u_4(1+S_C) - \beta_1(1+S_C)D \quad (7)$$

$$\bar{W}^2u_2' = u_2^2(1+S_C)^2 + u_2u_1(1+S_C)^2 + u_2u_3(1+S_C) + u_2u_4(1+S_C) + \beta_2(1+S_C)D \quad (8)$$

$$\bar{W}^2u_3' = u_3^2 + u_3u_1(1+S_C) + u_3u_2(1+S_C) + u_3u_4 + \beta_1(1+S_C)D \quad (9)$$

$$\bar{W}^2u_4' = u_4^2 + u_4u_1(1+S_C) + u_4u_2(1+S_C) + u_4u_3 - \beta_2(1+S_C)D \quad (10)$$

The cultural trait bias model can be similarly described.

S3. Simulation details

S3.1 Simulation of heterozygozity change

With these simulations, we aimed to demonstrate the potential effect on the genome of gc-pseudolinkage between a neutral genetic locus and a cultural trait under selection, and to compare this to the effect of physical linkage between neutral and selected genetic loci. We simulated a population of N haploid individuals who were characterised by five genetic loci with one focal locus at map position 0 and four linked loci. Two of these are on either side of locus 0 at a recombination distance $r = 0.001$ and two at $r = 0.2$. In the gene-culture simulations individuals also possessed a cultural trait.

Genetic linkage Simulations consist of two phases. In the first phase, all loci were selectively neutral. In each simulation run, parents were chosen at random from the population, formed into families and offspring were created, inheriting the full genome of one parent. Recombination occurred between parental genomes at a particular crossover point with a probability determined by distance from the focal locus. Mutation occurred at each locus with a probability $\mu = 0.0001$. Mutation introduced a new allelic type. This phase of the simulation was run to a steady state for $5N$ generations ensuring that the heterozygosity at each locus reached the expected neutral heterozygosity at freely recombining loci.

In the second phase, a single newborn was chosen at random from the population of newborns and an allele with fitness coefficient $S_A = 0.1$ was introduced at locus 0. Parents were then chosen with a probability weighted by fitness determined by the allele at locus 0. Recombination proceeded as in the first phase and mutation proceeded as in the first phase. The favored allele, A, was tracked in frequency until fixation or loss (or 2N generations if not yet fixed). Heterozygosity at each locus was calculated for all loci for all successful fixations. Results shown in the main text are for successful fixations only, with at least 200 fixations shown per condition.

Gc-pseudolinkage The first phase of the simulations ran as above for the case of genetic linkage. However, individuals were also characterised by a cultural trait which was linked to locus 0 according to Table 2 (affinity bias columns) with $\beta_1 = \beta_2 = \beta$.

In the second phase of this simulation, a single newborn was chosen at random from the population and a cultural trait with fitness coefficient $S_C = 0.1$ was introduced which was gc-pseudolinked to locus 0. Parents were then chosen with a probability weighted by fitness determined by the cultural trait. Note that in this case, locus 0 was neutral in terms of its direct effects on biological fitness. Recombination proceeded as in the first phase and mutation proceeded as in the first phase. The favored cultural trait was tracked in

frequency until fixation or loss (or 2N generations if not yet fixed). Heterozygosity was calculated for all loci for all successful fixations for main text Fig.3. Homozygosity values H_1 and H_2, as outlined in the main text were calculated for main text Fig.4. Results shown in main text Fig.3 and Fig.4 show successful fixations only, with at least 200 fixations shown per condition.

S3.2 Simulation of selective interference

We simulated a population of N individuals who were characterised by one genetic locus under natural selection (selection coefficient: S_A) and one cultural trait that also contributed to biological fitness (selection coefficient: S_C) according to main text equations (5)-(8). Each simulation time step involved one generation in which individuals were chosen as parents with probability weighted by individual fitness and formed into N pairs. N offspring were created from these families according to main text Table 2, either affinity bias or cultural trait bias. These N offspring then replaced the full population of parents.

For each independent simulation run, the initial frequency of the beneficial cultural background C was 0.5. Thus, 50% of the population had the beneficial cultural trait C and 50% had the ancestral cultural trait c. We then introduced a novel A allele at the genetic locus of one individual uniformly randomly selected from the population. This means that the beneficial mutation was introduced at a frequency of $1/N$ on either cultural background with equal probability.

We tracked the frequency of the beneficial allele and ran the simulation until the allele fixed or went extinct. The number of generations taken for the A allele to fix or go extinct was recorded and the number of generations for the A allele to fix, conditional on fixation, was recorded. The mean fixation time are presented relative to those found for an allele evolving from similar starting conditions in the absence of interference (main text Fig. 5). The number of fixations as a proportion of all simulations was also recorded and reported relative to the case of no interference.

We ran a sufficient number of independent simulations to provide at least 9,000 fixations for each parameter combination. Parameter values are provided in the figure captions.

S4. Full mating table

Below is an extended version of the mating table (Table 2) which was condensed in the main text. This version is included to ease comparison with existing similar models.

Family	Family frequency	Affinity bias: Offspring				Cultural trait bias: Offspring			
		AC	aC	Ac	ac	AC	aC	Ac	ac
AC×AC	$f_1 = x^2Q^2$	1	0	0	0	1	0	0	0
AC×aC	$f_2 = x^2Q(1-Q)$	0.5	0.5	0	0	0.5	0.5	0	0
AC×Ac	$f_3 = x(1-x)QR$	0.5	0	0.5	0	γ_1	0	1-γ_1	0
AC×ac	$f_4 = x(1-x)Q(1-R)$	$\frac{1-\beta_1}{2}$	$\frac{\beta_2}{2}$	$\frac{\beta_1}{2}$	$\frac{1-\beta_2}{2}$	$\frac{\gamma_1}{2}$	$\frac{\gamma_2}{2}$	$\frac{1-\gamma_1}{2}$	$\frac{1-\gamma_2}{2}$
aC×AC	$f_2 = x^2Q(1-Q)$	0.5	0.5	0	0	0.5	0.5	0	0
aC×aC	$f_5 = x^2(1-Q)^2$	0	1	0	0	0	1	0	0
aC×Ac	$f_6 = x(1-x)(1-Q)R$	$\frac{\beta_1}{2}$	$\frac{1-\beta_2}{2}$	$\frac{1-\beta_1}{2}$	$\frac{\beta_2}{2}$	$\frac{\gamma_1}{2}$	$\frac{\gamma_2}{2}$	$\frac{1-\gamma_1}{2}$	$\frac{1-\gamma_2}{2}$
aC×ac	$f_7 = x(1-x)(1-Q)(1-R)$	0	0.5	0	0.5	0	γ_2	0	$1-\gamma_2$
Ac×AC	$f_3 = x(1-x)QR$	0.5	0	0.5	0	γ_1	0	1-γ_1	0
Ac×aC	$f_6 = x(1-x)(1-Q)R$	$\frac{\beta_1}{2}$	$\frac{1-\beta_2}{2}$	$\frac{1-\beta_1}{2}$	$\frac{\beta_2}{2}$	$\frac{\gamma_1}{2}$	$\frac{\gamma_2}{2}$	$\frac{1-\gamma_1}{2}$	$\frac{1-\gamma_2}{2}$
Ac×Ac	$f_8 = (1-x)^2R^2$	0	0	1	0	0	0	1	0
Ac×ac	$f_9 = (1-x)^2R(1-R)$	0	0	0.5	0.5	0	0	0.5	0.5
ac×AC	$f_4 = x(1-x)Q(1-R)$	$\frac{1-\beta_1}{2}$	$\frac{\beta_2}{2}$	$\frac{\beta_1}{2}$	$\frac{1-\beta_2}{2}$	$\frac{\gamma_1}{2}$	$\frac{\gamma_2}{2}$	$\frac{1-\gamma_1}{2}$	$\frac{1-\gamma_2}{2}$
ac×aC	$f_7 = x(1-x)(1-Q)(1-R)$	0	0.5	0	0.5	0	γ_2	0	$1-\gamma_2$
ac×Ac	$f_9 = (1-x)^2R(1-R)$	0	0	0.5	0.5	0	0	0.5	0.5
ac×ac	$f_{10} = (1-x)^2(1-R)^2$	0	0	0	1	0	0	0	1

Table 3: Possible families (including the cultural trait possessed by the parents), their frequencies, and the frequencies of the next generation. The columns labelled ‘Affinity bias’ show the phenogenotypes in offspring in the case where β_1 is a tendency shown by A offspring to copy a different-genome parent, and β_2 is a tendency shown by a individuals to copy a different-genome parent. The columns labelled ‘Cultural trait bias’ show the same for the case where where γ_1 is the bias shown by A offspring towards ($\gamma_1 > 0.5$) or against ($\gamma_1 < 0.5$) the C cultural trait, and γ_2 is the bias shown by a individuals towards ($\gamma_2 > 0.5$) or against ($\gamma_2 < 0.5$) the C cultural trait.

References

M. W. Feldman and L. L. Cavalli-Sforza. Cultural and biological evolutionary processes: Gene-culture disequilibrium. Proceedings of the National Academy of Sciences, 81:1604–1607, 1984. ISSN 0027-8424. doi: 10.1073/pnas.81.5.1604.

J. Maynard Smith and John Haigh. The hitch-hiking effect of a favourable gene. Genetical Research, 23(1): 23–35, 1974. ISSN 14695073. doi: 10.1007/978-1-4613-3924-37.
